# Perceived Neighborhood Environments and Cardiovascular Risk Among Older Adults: The Role of Cognitive Activities

**DOI:** 10.1093/geroni/igaf122.2892

**Published:** 2025-12-31

**Authors:** Jeein Law (Jang)

**Affiliations:** University of Massachusetts Boston, Boston, Massachusetts, United States

## Abstract

Cardiovascular disease (CVD) is the leading cause of death in the United States, contributing to significant disability and healthcare costs. The neighborhood environment plays a critical role in cardiovascular health, particularly for older adults aging in place. Prior research has shown that higher physical disorder is associated with greater CVD risk, while greater social cohesion is associated with lower CVD risk. However, the role of cognitive activities as a moderator of these relationships, as well as the influence of subjective perceptions of neighborhood characteristics (PNC) on CVD rather than objective indicators, remains underexplored. Using pooled data from the 2016-2018 Health and Retirement Study (n = 6,248), logistic regression examined the likelihood of CVD based on PNC, adjusting for sociodemographic and health-related factors. CVD was defined as a self-reported diagnosis of heart-related diseases (e.g., heart attack, coronary heart disease, angina). Neighborhood cohesion and disorder were assessed based on perceptions of safety, trust, neighbor support, and signs of neglect or disrepair. Cognitive activities included reading, playing word or card games, and using a computer. Results showed that higher social cohesion was associated with a lower likelihood of CVD, while higher physical disorder was linked to greater CVD risk. Additionally, cognitive activity engagement weakened the positive association between physical disorder and CVD risk. These findings highlight the need for policies promoting cognitive engagement among older adults, particularly in disadvantaged neighborhoods. Expanding community-based programs that provide access to cognitive activities, such as libraries, senior centers, and digital literacy initiatives, could serve as protective health interventions.

